# Co contamination of Si: Plating & aging

**DOI:** 10.1016/j.heliyon.2024.e32530

**Published:** 2024-06-08

**Authors:** Jintao Wang, Dingning Ke, Feng Tian, Li Xiong, Hongtao Chen, Mingyu Li

**Affiliations:** a Education Center of Experiments and Innovations, Harbin Institute of Technology (Shenzhen), 518055, China; b Sauvage Laboratory for Smart Materials, Harbin Institute of Technology (Shenzhen), Shenzhen, 518055, China; c School of Material Science and Engineering, Harbin Institute of Technology (Weihai), Weihai, 264209, China

**Keywords:** Pulse plating, DFT, Electronic packaging, Advanced Packaging

## Abstract

Cobalt has emerged as a vital material in 10 nm technology for localized interconnect layers, potentially offering a compelling alternative to Cu-based interconnects. In this study, we subjected the contamination arising from the presence of cobalt atoms in silicon to comprehensive investigation, employing electron transmission electron microscopy (TEM) observations in conjunction with first-principles calculations. The results show that a dense CoSi layer with a thickness of a few nanometers is formed at the interface of cobalt and Si. The CoSi layer blocks the diffusion of Co atoms into Si. This is due to the semiconducting nature of the covalent bond formed between Co and Si, leading to the emergence of a forbidden zone at the Co/CoSi interface. The diffusion of Co into CoSi is governed by the atomic exchange mechanism, however, the local distortion of the periodic atomic potential due to the presence of the forbidden zone at the Co/CoSi interface hinders the diffusion of Co into Si. Therefore, the deposition of a Co metal layer on a Si chip does not require an additional barrier layer.

## Highlights

• We have employed the pulse plating technique to effectively deposit cobalt nanocrystals onto silicon wafers.
• At the cobalt-silicon interface, we observed the formation of a dense CoSi layer with a thickness of a few nanometers.
• As a result of the CoSi layer's hindrance to the diffusion of cobalt atoms, cobalt primarily accumulated at the interface and displayed limited diffusion into the silicon substrate.
• The diffusion of cobalt within silicon predominantly adheres to the vacancy diffusion mechanism, whereas cobalt diffusion into CoSi is primarily governed by the atomic exchange mechanism. Notably, the Co–CoSi interface experiences periodic atomic potential energy distortions, imposing a significant barrier to further cobalt diffusion.

## Graphical abstract

**Figure:**
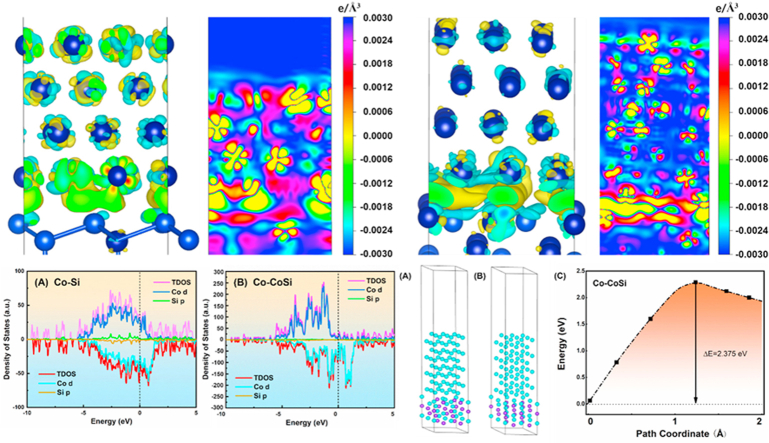

## 1 Introduction

Copper interconnects have served as the cornerstone of Back End of Line technology ever since their incorporation into Very Large Scale Integrated (VLSI) circuit products in the late 1990s. Nevertheless, as the dimensions of copper interconnects continue to decrease, the consequences of grain boundary scattering and surface scattering are becoming more pronounced. This leads to a notable escalation in the resistance encountered by copper interconnects [[1], [2], [3], [4]]. However, as interconnect sizes continue to shrink, the effects of grain boundary scattering and surface scattering of Cu are increasing, leading to a significant increase in the resistance of Cu interconnects. In addition, since Cu is highly reactive, contamination by physical diffusion from Cu to silicon (Si) and chemical combinations between Cu and Si are commonly observed and are detrimental to Si semiconductor devices [5]. Therefore Cu interconnects require a barrier and adhesion layer, usually TaN, at the Cu/Si interface. When the Cu interconnect gets smaller, the tantalum nitride lining remains relatively thick, and the lining occupies the space for the Cu, which not only leads to an increase in resistance and capacitance of the line and vias, but also to an increase in the resistance and capacitance of the line and the vias when filled with Poor filling of Damascene trenches below 30 nm with copper occurs due to the limitations of the Physical Vapor Deposition (PVD) process, resulting in the creation of metal voids that further increase line resistance, causing an increase in resistor-capacitor delays and severely slowing down semiconductor components. Alternative conductors such as Co, Ru, Rh, etc. have been proposed as viable substitutes for the smallest pitch interconnect layers to achieve even lower resistances, tighter resistance distribution and superior reliability [[6], [7], [8], [9], [10]].

**Fig. 1:**
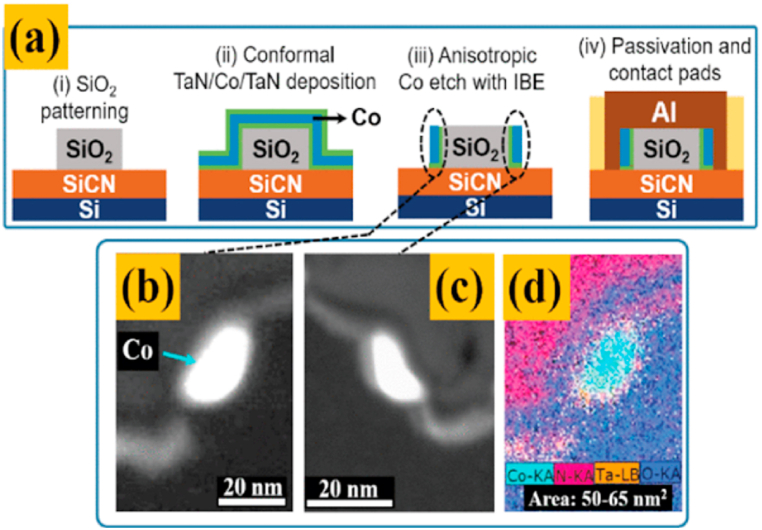
(a) Schematic representation of the wire fabrication process (b), (c) Cross-section HAADF-STEM images across the wires. (d) Elemental mapping (EELS) of Co, Ta, N, O across the wires (adopted from Ref. [3], Reproduced with permission Copyright©2018, IEEE).

To address the scaling challenge, Intel has introduced a new metallization solution for 10 nm nodes to meet the reliability and performance needs of the EM local interconnect layer. Cobalt is the metal introduced in 10 nm technology for local interconnect layers (Fig. 1) [[11], [12], [13], [14], [15], [16]].Complete cobalt integration can be considered. However, there is still a lack of research on the contamination and effects of Co atoms in Si. In addition, deposition schemes for Co on Si are in an early state of research, and interconnect plating solutions using cobalt seed crystals must etch the natural oxides that are formed instantaneously during air disruption while preserving the metal seed crystals underneath.

Therefore, we carried out a study of the cobalt-filled Bosch (BEOL) process, where well-bonded seed crystals were fabricated on Si wafers by vapor deposition, and Co nanocrystals well-coated on Si wafers were achieved using a pulse plating process. The contamination of Co atoms in Si was investigated using electron transmission electron microscopy observations and first principles calculations.

## 2 Experimental and calculations

The seed layer was first fabricated on a silicon wafer by a vacuum thermal evaporation process (INFICON, SOC-310) with a thickness of 20 nm, a speed of 0.2 nm/s, an evaporation temperature of 1600 °C, an operating voltage of 5 V, and a current of 80 A. Electron microscopic observations of the seed layer were carried out by electron scanning microscopy and atomic force microscopy.

Assuming that the evaporation source is an ideal point source, it can be determined by Gauss's theorem that, with the point source as the center of the sphere, the deposition rate at any point on the spherical shell of the same radius is equal; whereas the deposition rate at two points on the spherical shell of different radii is unequal, and the deposition rate at the sphere of large radius is small. Since the coated substrate is a flat surface and has a certain size. Different points on the substrate are located in the area of the microelement distance from the evaporation source, as well as the microelement and the evaporation source line and the microelement plane between the clip. The angles are also different, which results in a certain thickness difference on the substrate. This non-uniformity of thickness has a great impact on the consistency of the post-plating properties.

**Fig. 2:**
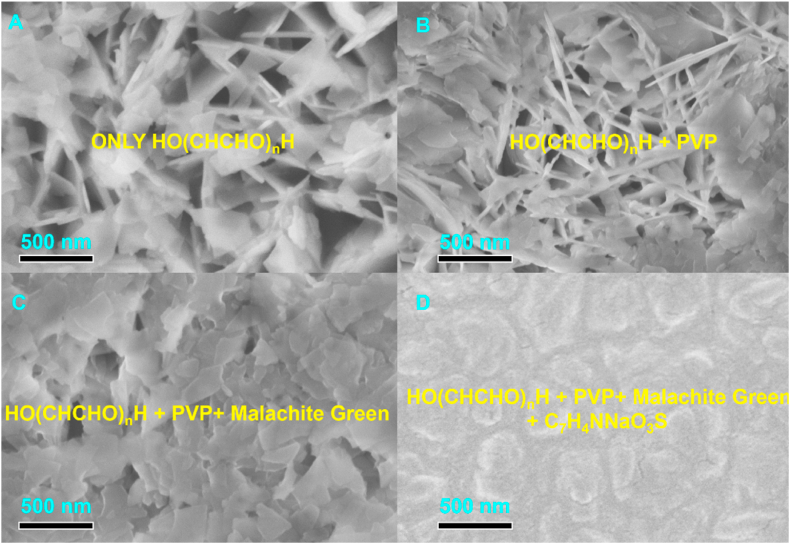
SEM images of Pulse Plated Co with Different additives.(A) only polyethylene glycol (B) polyethylene glycol and PVP (C) polyethylene glycol, PVP and Malachite Green (D) polyethylene glycol, PVP and Malachite Green, C_7_H_4_NNaO_3_S.

Therefore, in the vacuum evaporation coating process, we make a planetary rotation of the silicon substrate to improve the uniformity. In fact, the substrate always runs on the same ball shell (Fig. 2A).

The unidirectional time was 0.3 ms and the turn-off time was 0.1 ms under a pulsed current with a current density of 0.8 A/dm^2^. The positive electrode was a graphite electrode, and the negative electrode was a Co-plated silicon wafer, with a stirring rate of 700 r/s and a plating temperature of 40 °C. The electroplating solution was a sulfamate electrode with an electrolyte of 1.5 m/s.

**Fig. 3:**
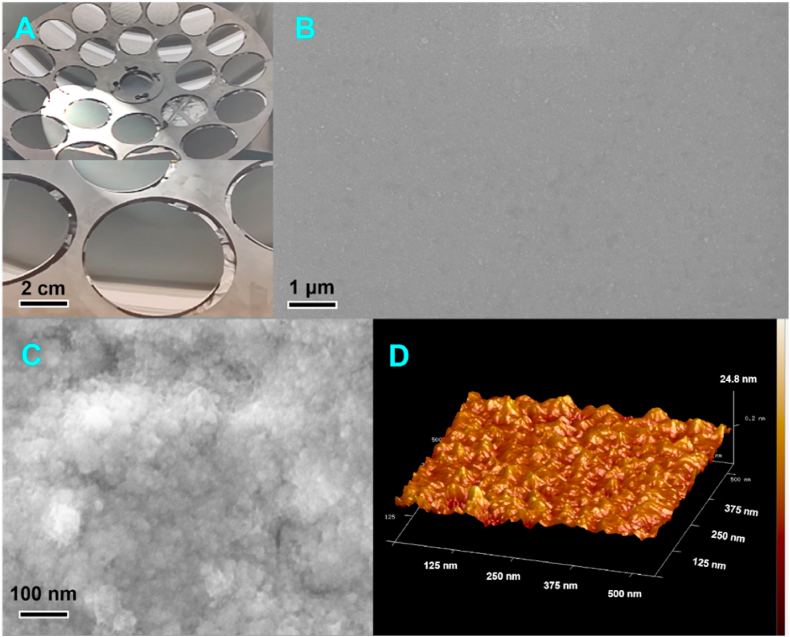
SEM images of Si substrate after vapor deposition of Co seed layers (A) Vacuum Thermal Evaporation Process (B) The surface morphology of the cobalt metal film (C) The surface morphology of the cobalt metal film (D) Surface roughness of cobalt film obtained by atomic force microscopy.

The base plating solution we chose was cobalt sulfamate solution with 2-mercapto-5-benzimidazole sulfonic acid and sodium dodecyl sulfonate (both as anionic surfactants), and the surface morphology of pulse electroplated cobalt coatings with different additive compositions was observed and analyzed by scanning electron microscope, and the results are shown in Fig. 2. The results are shown in Fig. 3. From Fig. 2, it can be seen that polyethylene glycol can make the precipitation reaction more intense, obtain the pinhole-like morphology and promote the deposition of Co. The pinhole morphology was improved by adding Polyvinyl pyrrolidone（PVP）. According to the literature, after adding the reaction inhibitor malachite green, malachite green can effectively inhibit the deposition of cobalt, control the rate of electrochemical reaction of cobalt at the cathode, eliminate the concentration polarization of cobalt in the solution, so that cobalt can be uniformly nucleated and grown. After adding wetting agent sodium saccharin in the plating solution, it can effectively slow down the growth rate of grain, reduce the concentration polarization of the plating solution, make the surface of the plating layer more uniform, dense, flat, without obvious pinholes and burrs [12,17].

The addition of **Saccharin sodium** (**C**_**7**_**H**_**4**_**NNaO**_**3**_**S**) improves the hydrophilicity of the layer and facilitates the removal of air bubbles, thus improving the quality of the cobalt layer. The addition of a wetting agent to the base plating solution effectively improves the morphology of the cobalt layer deposited. The wetting agent molecules work synergistically with the Cl^−^ ions to form an inner-sphere electron transfer model that accelerates the reduction of cobalt ions, leading to denser crystallization [18,19].

Aging experiments were performed on cobalt-plated Si wafers after electroplating, which were heated in vacuum at 150 °C for 30 days, and FIB slices were taken on the cross section of the wafers to observe the contamination of Si wafers by Co atoms. DFT calculations were also performed to study the contamination behavior of Co atoms in Si. The morphology of the plated layer was observed by atomic force microscopy and transmission electron microscopy.

The diffusion energy barrier can be defined as the minimum energy required to relocate an individual atom from its initial site to a destination site without disturbing the surrounding atoms [15,31]. In this study, we employ the Transition State Search (TSS) method, available within the CASTEP module of MS. The fundamental principle behind this method involves determining the minimal migration energy and pathway by constructing two models: one representing the initial state and the other depicting the state after diffusion. This approach involves isolating the diffusing copper atom from the other atoms based on proximity principles and geometric relaxation results. The exchange functional used is the generalized-gradient approximation (GGA) based on the Perdew-Burke-Ernzerhof (PBE) formulation. Convergence criteria for energy, maximum force, maximum stress, and maximum displacement are established at 1.0 × 10^−5^ eV/atom, 0.03 eV/Å, 0.05 GPa, and 0.001 Å, respectively. The Broyden–Fletcher–Goldfarb–Shanno (BFGS) method is employed as the optimization algorithm. In terms of electronic settings, an energy cutoff of 400 eV is applied, the self-consistent field (SCF) tolerance is set at 1.0 × 10^−6^ eV/atom, and Monkhorst-Pack k-points with a grid of 4 × 4 × 1 are utilized. Ultrasoft pseudopotentials are employed, and the pseudopotential representation is utilized in reciprocal space. Fig. 7(a) is Co (0 0 1)/Si (1 1 1) interface built in MS. A vacuum layer of 15 Å is added perpendicular to the sheet to avoid artificial interaction between periodic images. Fig. 9(a) illustrates the crystal structures of Co as incorporated into the simulation software, serving as the foundation for the subsequent Co/CoSi interface heterojunction model. The optimization of Co/Si interface structures involves Brillouin zone integration employing Gamma-only grids. All structures undergo relaxation until the residual forces acting on the atoms diminish to below 0.05 eV/Å. The transfer barrier, denoted as ΔE, is calculated as the difference between the energies in the transition state (E(TS)) and the initial state (E(IS). For the investigation of diffusion, a Co/CoSi interface heterojunction model is employed, depicted in Fig. 9(b), comprising 88 Co atoms and 16 Si atoms. The construction of this heterojunction structure utilizes a four-layered 3 × 3 Co (001) supercell and a four-layered 2 × 2 CoSi (111) supercell, effectively accommodating the mismatch in lattice constants between Co and Si. The lattice constants for the Co/CoSi heterojunction structure are as follows: a = 7.6759 Å, b = 7.6759 Å, and c = 40.5641 Å. The lattice mismatch between the Co and CoSi supercells equates to 5.7 % [[20], [21], [22], [23]].

## 3 Results and analysis

The micro-morphology of the cobalt metal films deposited on silicon substrate was characterized using field emission electron microscopy, as shown in Fig. 3. Fig. 3(B) shows the surface morphology of the cobalt metal film, which can be seen that the surface of the cobalt metal film deposited on the silicon substrate is relatively dense, without the presence of holes and cracks, and the surface of the film is a continuous peak-like morphology (Fig. 3), which indicates that the film grows in the form of an island-like growth mode.

The forward and reverse pulse plating method is an effective technique for achieving bottom-up void-free filling. The presence of a reverse pulse current allows for a small amount of stripping of the surface plating, which promotes the formation of a flatter and denser plating layer. Additionally, the reverse pulse increases the ion transport rate, which leads to a higher rate of metal deposition at the bottom of the micro-via. This technique also eliminates hydrogen embrittlement, reduces internal stresses, and increases the bonding force between the coating and the substrate. By controlling the electrochemical reaction rate of cobalt at the cathode and eliminating the concentration polarization of cobalt in solution, cobalt can be uniformly nucleated and grown.

Through the electron microscope photographs, obvious island-like expansion of the plating layer can be seen, indicating that internal stresses are generated by the lateral growth of grain aggregates or some of them from different nucleation centers during the formation of crystallization of electrodeposited Co metal. When dislocations occur because of grain aggregation or other growth processes leading to directional alignment, the stress field around the dislocations increases, which promotes the increase of internal stress.

**Fig. 4:**
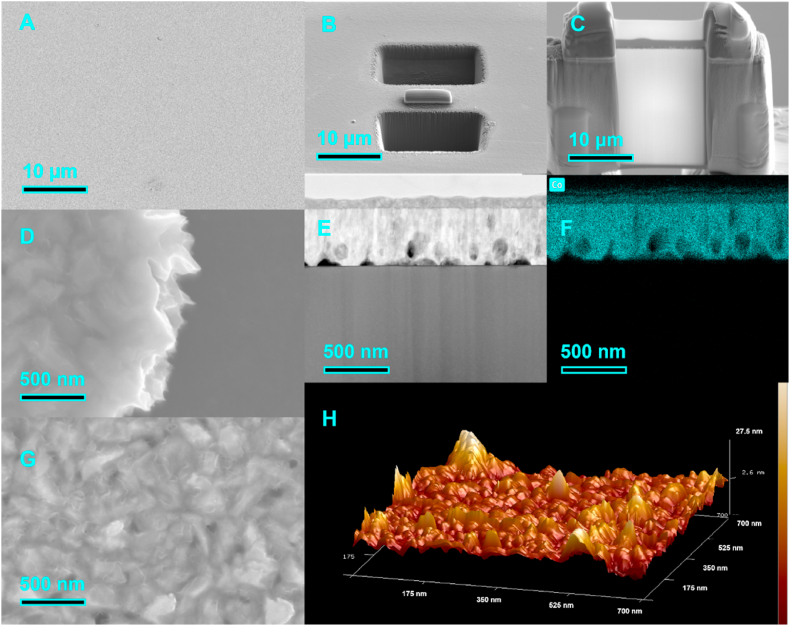
FIB (focused ion beam system) section of surface Co layer and Si substrate after electroplating (A) Co surface SEM images (B) FIB slicing process (C) Cross section obtained by FIB slicing (D) Interface between Si substrate and Co layer (E) Interface between Si substrate and Co layers (F) EDX result of the interface between Si substrate and Co layers (G) SEM image of Co layer (H) Surface roughness of cobalt film after electroplating obtained by atomic force microscopy.

**Fig. 5:**
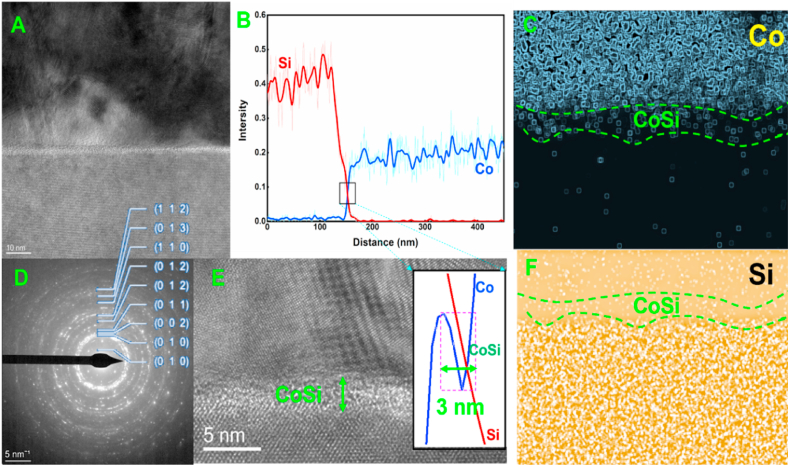
(A) TEM images of the interface between Si substrate and Co layer (B) EDX result of the interface between Si substrate and Co layer (C) Mapping of Co element in the interface between Si substrate and Co layer (D) Polycrystalline diffraction ring of Co (E) TEM images and EDX result of the CoSi. (F) Mapping of Si element in the interface between Si substrate and Co laye.

From Fig. 4, the surface of the Co layer obtained by electroplating is smooth and flat, the Co grain size is about 500 nm, the thickness of the layer is about 1 μm, and with the help of AFM, the surface undulation of the layer can be seen to be about 20 nm. EDX testing confirmed the chemical composition of the Co coating. Using an electron transmission microscope, we observed the Co/Si interface after 30 days of aging at 150 °C. The Co/Si interface was observed by an electron transmission microscope. After EDX scanning, the Co atom concentration decreases rapidly along the Co/Si interface and does not diffuse onto the interior of the Si layer.The diffusion distance of the Co atoms is only about 5 nm, and through the diffraction ring, we can see that the Co layer consists of a large number of nanocrystals, and therefore the diffusion of Co should be dominated by the diffusion of the grain boundaries in the early stage. During the deposition of Co onto the Si surface, Co atoms tend to climb up to build up the plating layer. Interestingly, after aging, Co atoms start to accumulate at the Co/Si interface (Fig. 5-e). From the EDX results, there is an enriched zone of about several nanometers thick for Co atoms at the interface. In addition, the transport of Si atoms through the Co layer can be observed, which leads to a gradual increase in the total Si content in the Co film. Similar to the accumulation of Co atoms in the interfacial layer, Si accumulation occurs at the Si/Co interface. We have observed the atomic arrangement at the interface with the aid of transmission electron microscopy, and it can be seen that immediately after the deposition of Co onto the Si substrate, Co atoms are bound to the surface of Si through an exchange mechanism, and diffusion of Si atoms into the Co grains also occurs [24].

For embedded Si the Co atoms diffuse and may form small clusters in the substrate. Two mechanisms by which the embedded atoms can diffuse are (1) diffusion by exchange of atoms and (2) interstitial diffusion. For the atom exchange mechanism, when Co atoms are adsorbed by Si atoms, the Co atoms and Si atoms first exchange positions, and then the newly moved Si adsorbed atoms diffuse on the Co surface and then reincorporate into the interface through a new exchange process. This is consistent with our observation that diffusion of both Co and Si atoms into each other's lattice occurs almost symmetrically.

**Fig. 6:**
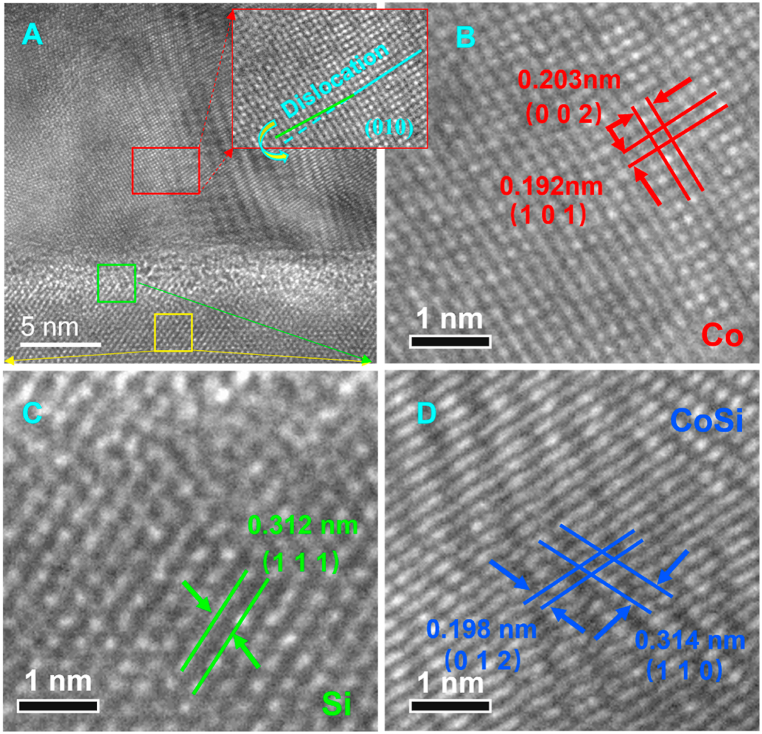
(A) TEM images of the interface between Si substrate and Co layer (B) TEM image of the Co (C) TEM image of the Si (D) TEM of the CoSi.

According to the experimental results we observe the existence of CoSi layer, which means that the exchange atom diffusion mechanism exists and the CoSi layer is the result of Co diffusion into Si. In contrast to interstitial diffusion, in the atom exchange diffusion mechanism, Co atoms must cross the CoSi interface before entering the Si lattice. Surface interstitial diffusion is often accompanied by the breaking of existing Co–Co bonds, Si–Si bonds, and interfacial Co–Si bonds, as well as the formation of new Co–Si bonds in the Si lattice, which leads to an increased energy barrier for surface interstitial diffusion. In fact, the free Co dissolved in the Si matrix is not in the atomic state but in the ionic state and is even capable of covalent interactions, as shown in Fig. 6.

**Fig. 7:**
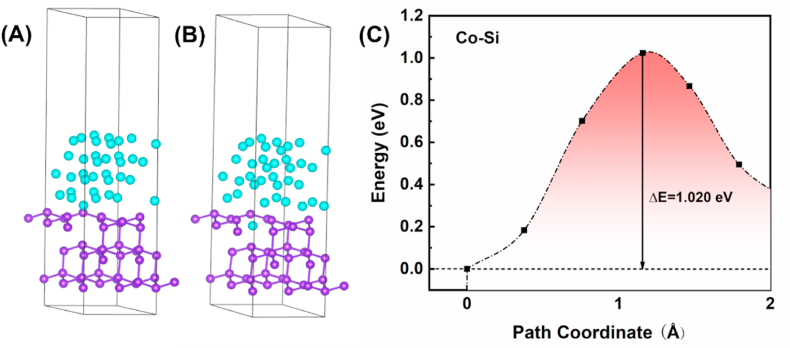
(A) Model of the Co(001)/Si(111) interface. (B) The schematic diagram of interstitial diffusion of Co in Si surface (C) the diffusion energy barrier when vacancy at top site.

We have calculated the diffusion energy barriers for diffusion of Co atoms into Si for both mechanisms with the help of the first nature principle, and it is obvious to look for ways to have higher potential barriers. The diffusion energy barrier is expressed as the energy difference between the peak energy and the initial energy of the total system during the movement of Co atoms towards the destination. In addition we need to investigate the reason why this mechanism has a higher energy barrier by calculating the electron density difference and DOS.

**Fig. 8:**
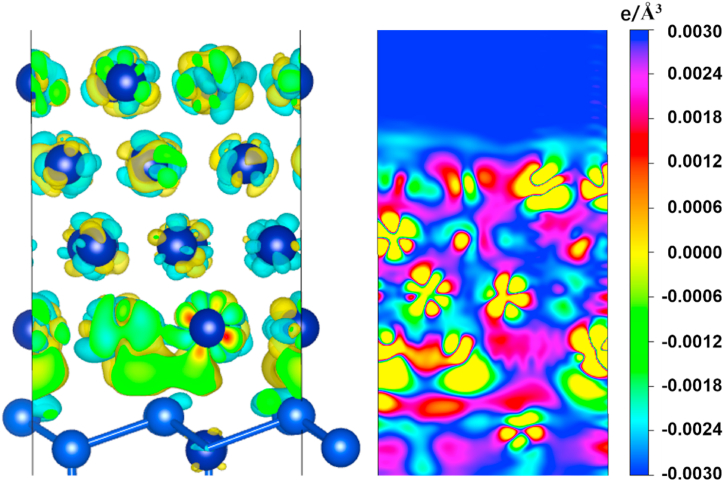
The electron density difference calculation of Co/Si (111) heterojunction.

Calculations indicate that Co atoms have the smallest diffusion energy barrier when there are vacancies in the bridge sites on the Si surface, as shown in Fig. 7(c). The peak in the graph implies that the maximum energy difference for realizing the diffusion is 1.020 eV. These results suggest that the possibility of Co diffusion into Si is high. From the DOS calculations, Co diffuses into the vacancies in Si and then binds to the neighboring Si atoms with covalent bonds. The hybridization of Co-d and Si-p orbitals also confirms the existence of a strong covalent bond between Co and the Si acceptor. When Co atoms diffuse into the vacancies in the Si surface in large quantities, the Si atoms in the surface layer form a chemical bond with the Co atoms occupying the vacancies. This bond hinders the further diffusion of subsequent Co atoms. Additionally, the presence of covalent bonds prevents Co atoms from leaving the occupied vacancies to penetrate deeper into the Si. This means that CoSi compounds prevent Co atoms from further contaminating the Si and also prevent Si atoms from entering the Co layer（Fig. 8,Fig. 10）.

**Fig. 9:**
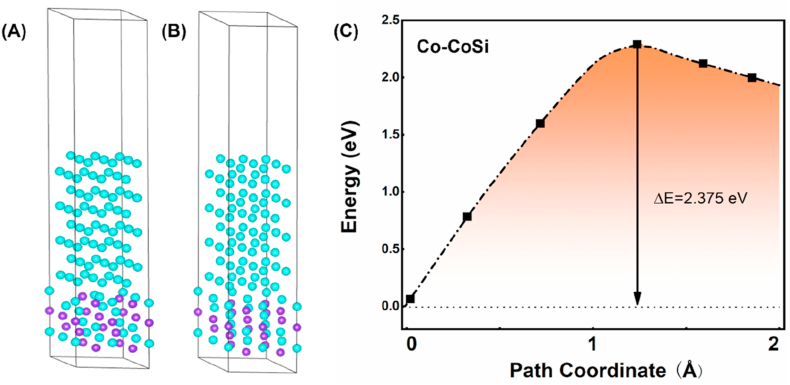
(A) Model of the Co(001)/CoSi(111) interface. (B) The schematic diagram of diffusion of Co in CoSi surface through the atomic exchange mechanism (C) the diffusion energy barrier when Diffusion through the atomic exchange mechanism.

**Fig. 10:**
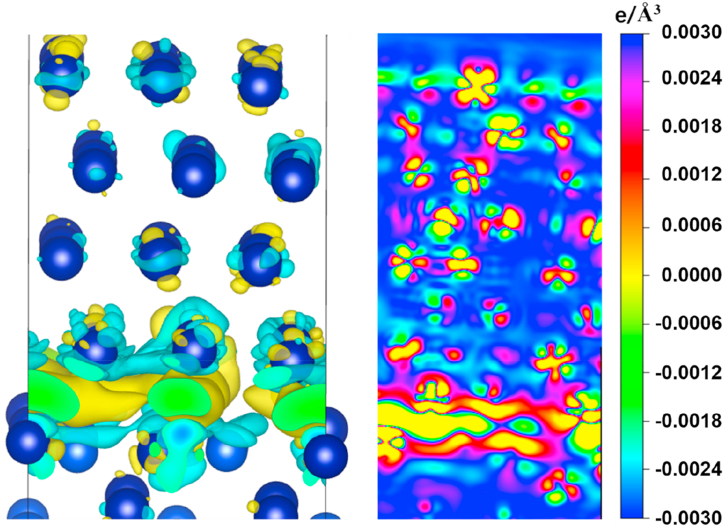
The electron density difference calculation of Co/CoSi (111) heterojunction.

According to differential charge calculations, Co atoms are energetically more favorable when surrounded by four Si atoms after diffusion with the help of vacancies. A vacancy occupying one of the nearest neighbor positions will reduce coordination and increase the total free energy of the configuration. Thus the diffusion of Co atoms into Si relies on the interstitial spaces between Si atoms to take place rather than occupying vacancies. The nature of the interaction between the interstitial and the embedded Co atoms is repulsive due to the transfer of electrons from the Co atoms into the Si atoms. When a Co atom enters a vacancy in Si, the d-orbital of the Co atom tends to form a hybridization with the p-orbital of the Si atom. This process occurs through the loss of valence band electrons from the Co atom to the Si atom. Additionally, a region of free electron enrichment occurs at the interface between Co and Si, which indicates the attraction of the valence electrons of Co atoms by Si atoms. The enrichment of free electrons increases the binding ability of Co to Si.

**Fig. 11:**
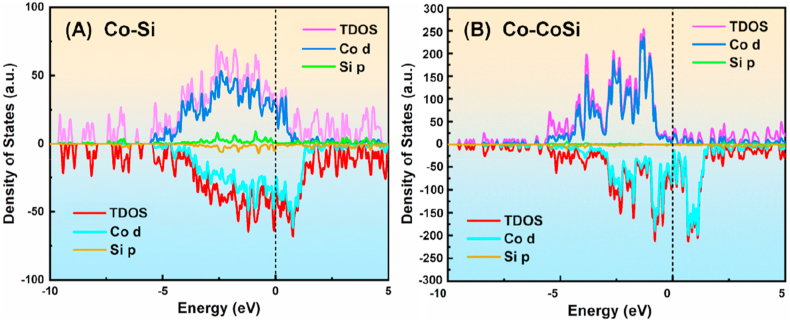
Energy band structure near the Fermi surface and the corresponding electronic energy state density profile （A）Co–Si interface (B) Co–CoSi interface.

In contrast to interstitial diffusion, the entry of Co into Si via an exchange diffusion mechanism requires prior access to the CoSi layer. The diffusion energy barrier required for Co to enter the CoSi layer is as high as 2.375 eV (Fig. 9C). DOS plots show that the electronic structures of CoSi and Co are similar in nature (Fig. 11). In both cases, the spin polarization is clear: relaxation of the self-filling interstitial leads to a symmetric configuration in which the interstitial-filling atoms push the two neighboring lattice sites outward. The presence of covalent bonding between Co and Si leads to a forbidden band at the interface. From the energy band structure near the Fermi surface and the corresponding electronic energy state density, CoSi also has semiconducting properties. The existence of the forbidden band at the Co/CoSi interface makes it difficult for Co atoms to enter into CoSi, and even more difficult to enter into the vacancies in Si. That is to say, the tunneling barrier at the interface leads to the local distortion of the periodic atomic potential, which hinders the further diffusion of Co atoms into Si [25].

In summary, during the seed layer fabrication process, Co atoms deposited on the surface of Si spontaneously diffuse into Si through the Si atomic interstitial. After Co atoms occupy the vacancies, the valence electrons of Co atoms are attracted by Si atoms, and the d orbitals of Co atoms form hybridization with the p orbitals of Si atoms. The Co/Si interface appears to be enriched in free electrons. The covalent bond between Co and Si forms, semiconducting properties appear, resulting in the appearance of forbidden bands at the Co/CoSi interface. The diffusion of Co into CoSi is dominated by the atomic exchange mechanism. However, due to the existence of forbidden bands at the Co/CoSi interface, local distortions of periodic atomic potentials impede the diffusion of Co into Si.

## 4 Conclusions

Effective deposition of cobalt (Co) onto silicon (Si) can be achieved through a two-step process involving the evaporation of a seed layer onto Si, followed by pulse plating.

Using electron transmission microscopy, we examined the Co/Si interface following 30 days of aging at 150 °C. The analysis revealed a rapid reduction in the concentration of Co atoms along the Co/Si interface, with no discernible diffusion into the Si layer. The diffusion distance covered by Co atoms was limited to approximately 5 nm.

Through first-principles calculations, we determined that the energy barrier for Co diffusion into Si is 1.020 eV, while the energy barrier for Co diffusion into CoSi is 2.375 eV. This discrepancy can be attributed to the semiconducting properties of CoSi. Additionally, the presence of forbidden bands at the Co/CoSi interface induces periodic atomic potential distortions, rendering the diffusion of Co into Si challenging.

## Data and code availability

Not Applicable.

## Ethical approval

Not Applicable.

## CRediT authorship contribution statement

**Jintao Wang:** Writing – original draft, Formal analysis. **Dingning Ke:** Methodology. **Feng Tian:** Software. **Li Xiong:** Formal analysis. **Hongtao Chen:** Writing – review & editing, Resources. **Mingyu Li:** Resources.

## Declaration of competing interest

The authors declare that they have no known competing financial interests or personal relationships that could have appeared to influence the work reported in this paper.
